# Hyperosmolarity of mouse urine confounds research in urinary tract infection

**DOI:** 10.1038/s41684-026-01727-4

**Published:** 2026-04-29

**Authors:** Kristian Stærk, Janni Søvsø Hjelmager, Rasmus Birkholm Grønnemose, Marie Lykke Bach, Jens Sivkær Pettersen, Thomas Emil Andersen

**Affiliations:** 1 Department of Clinical Microbiology, Odense University Hospital, Odense, Denmark; 2 Research Unit of Clinical Microbiology, University of Southern Denmark, Odense, Denmark; 3 Cardiovascular and Renal Research, Department of Molecular Medicine, University of Southern Denmark, Odense, Denmark; 4 Department of Biochemistry and Molecular Biology, University of Southern Denmark, Odense, Denmark

**Keywords:** Bacterial infection, Pathogens

## Abstract

Mice have been used as models of urinary tract infection (UTI) for decades and laid the basis for the fundamental understanding of UTI pathogenesis in humans. Here we report that the high urine osmolarity of mice impacts key aspects of *Escherichia coli* UTI pathogenesis and represents a confounder for the translation of results to humans.

## Abstract (web summary)

The authors report that the high urine osmolarity of mice impacts key aspects of *Escherichia coli* urinary tract infection (UTI), including bacterial filamentation and infectious potential, with important consequences for modeling human UTI in mice.

## Main

Urinary tract infections (UTIs) reduce the quality of life of millions and is a considerable cause of disease and death in vulnerable individuals^1^. Importantly, UTI is a major driver of antibiotic consumption and antibiotic resistance, thus emphasizing the need to understand UTI pathogenesis and develop new treatment strategies^2^. The mouse has for decades been regarded as a critical experimental model for deciphering basic UTI pathogenesis, due to its small size, ease of maintenance, low cost and amenability to genetic manipulation^3^. Nevertheless, it also has shortcomings as a model of human disease. In health science, the emphasis on mice is believed to be a contributor to the failure of drug and vaccine clinical trials^4,5^. Awareness of the limitations of this model is therefore critical in translational medical research.

Of relevance to the use of mice in UTI research is the unique capacity of mice to produce highly concentrated urine. Mice produce urine at concentrations far outside the range of humans and most other mammals^6^. It has remained unexplored how this fundamental physiological trait of mice impacts the transferability of UTI research to humans.

We hypothesized that the highly concentrated urine of mice affects bacterial growth fitness and furthermore is an inducer of bacterial filamentation, a key step in UTI pathogenesis that has been extensively studied in mice where filamentation is abundant^3,7,8^. Uropathogenic *Escherichia coli* (UPEC) elongates and forms filaments to some extent during growth in highly concentrated human urine, and during experimental infection of human bladder epithelial cells exposed to human urine^9,10^. Induction of the phenotype requires human urine at concentrations above a certain threshold and is moreover affected by surface growth and pH^9–11^. UPEC filaments have been observed in human urine from patients with UTI^12^, although our experience from routine inspection of human patient urine samples is that they are relatively rare, unless the patient is treated with filament-inducing antibiotics.

**Fig. 1:**
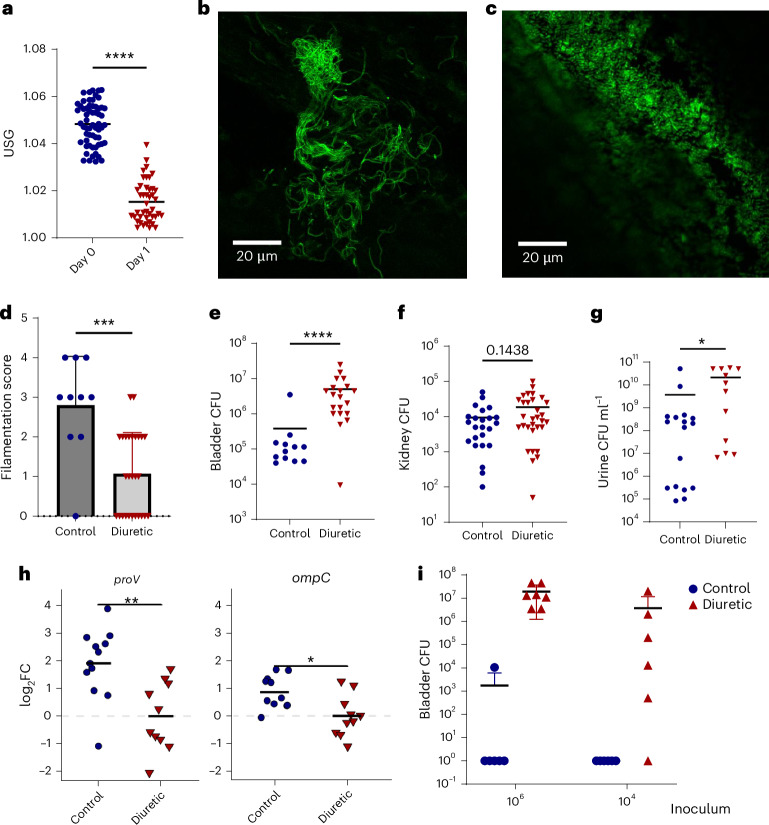
Mouse urine is an osmotic stressor on UPEC that promotes filamentation and reduces infectious capacity. **a**, Promoting water intake by feeding mice with sweetened water significantly reduced USG (equals density) from day 0 (baseline) to day 1 (24 h after giving sweetened water) (*n* = 64, *P* < 0.0001, Mann–Whitney test). **b**–**d**, Significantly higher level of UPEC filamentation was observed in mice with access to regular tap water (control; **b**) (*n* = 10) compared with mice with access to sweetened water (diuretic mice; **c**) (*n* = 27); results were quantified by manual counting using confocal laser scanning microscopy (**d**), with bars showing mean ± s.d. (*P* = 0.0003, Mann–Whitney test). **e**–**g**, The low USG in diuretic mice increased UPEC survival after 18 h of infection in the bladder (**e**) (*P* < 0.0001, Mann–Whitney test; control (*n* = 12), diuretic (*n* = 20)); in the urine (**g**) (*P* = 0.01, Mann–Whitney test; control (*n* = 17), diuretic (*n* = 12)); but CFUs were not significantly different in kidneys (**f**) (*P* = 0.14; control (*n* = 24), diuretic (*n* = 31)). **h**, Osmo-responsive genes *proV* and *ompC*, monitored by RT–qPCR were induced in control mice (*n* = 12) relative to diuretic mice (*n* = 10), (*P* < 0.01, Mann–Whitney test). **i**, Compared with control mice, diuretic mice were significantly more susceptible to infection at reduced infectious doses of 10^6^ CFU ml^−1^ (*P* < 0.001, Fisher’s exact test) and 10^4^ CFU ml^−1^ (*P* < 0.015, Fisher’s exact test). Horizontal bars represent means. ****P < 0.0001, ***P < 0.001, **P < 0.01, *P < 0.05.

To assess the effect of the high osmolarity of mouse urine on UPEC morphology and infectious potential, we established a natural diuresis mouse model by allowing animals access to sweetened water (tap water supplemented with 20% (w/v) glucose) 30 h before infection. Increased water intake reduces the urine concentration of mice—measured as urine specific gravity (USG, that is, density)—to levels comparable to those of humans (normal range 1.005–1.030) (Fig. 1a). Increased water intake did not significantly affect urine glucose levels, pH, the urine albumin-to-creatinine ratio or total urinary protein excretion; however, wide variability was observed in the urine albumin-to-creatinine ratio in diuretic mice (Supplementary Fig. 1).

Diuresis and control mice were then experimentally infected with the prototypical UPEC isolate UTI89 transformed with the green fluorescent protein-encoding plasmid pMAN01. The mice were euthanized after 18 h—a sweet spot for observing filaments in this model (which occur 12–20 h after infection)—and bacterial morphology was examined on splayed bladders by microscopy^7,13^. In control mice, extensive filamentation was observed, whereas bacteria remained mainly rod-shaped in diuresis mice (mean filamentation score of 2.80 and 1.07, respectively; *P* = 0.0003, Mann–Whitney test) (Fig. 1b–d). In 41% of diuresis mice, rod-shaped bacteria were exclusively observed without any detectable filamentation (Fig. 1b–d). Bacterial colony-forming units (CFUs) were significantly higher in the urine (*P* = 0.01, Mann–Whitney) and bladders (*P* < 0.0001, Mann–Whitney) of diuresis mice than in controls, but not different in kidneys (Fig. 1e–g). Reverse-transcription quantitative polymerase chain reaction (RT–qPCR) analysis indicated osmotic stress in bacteria from control mice compared with diuresis mice, based on increased expression of the central osmo-responsive gene *proV* from the osmo-inducible proU operon and *ompC* (Fig. 1h, mean log_2_ fold change (FC) of 1.90 and 0.85, respectively)^14,15^.

The higher bacterial burden in the diuresis mice led us to speculate that USG might also affect susceptibility to infection. Mice are intrinsically resistant to UTI and require high inocula of 10^8^–10^9^ CFU ml^−1^ for successful infection^3^. This dose is approximately a million times higher than that used in pigs, an alternative model animal that is naturally susceptible to UTI and produces urine with USG levels comparable to those of humans^16^. To test this hypothesis, groups of diuresis and control mice were inoculated with 10^6^ and 10^4^ CFU ml^−1^. At 10^6^ CFU ml^−1^, only one of six control animals (17%) became infected compared with seven of seven (100%) diuretic mice (*P* < 0.001, Fisher’s exact test) (Fig. 1i). Similarly, for 10^4^ CFU ml^−1^, all control animals (*n* = 6) resisted infection whereas diuresis mice remained highly susceptible, with five of six (83%) becoming successfully infected (*P* = 0.015). Of note, in the previous experiment, all mice from both groups inoculated with 10^9^ CFU ml^−1^ became infected (Fig. 1e). Taken together, these results suggest that lowering the USG of mice to values in the human range decreases the necessary infectious dose by 10,000-fold or more.

**Fig. 2:**
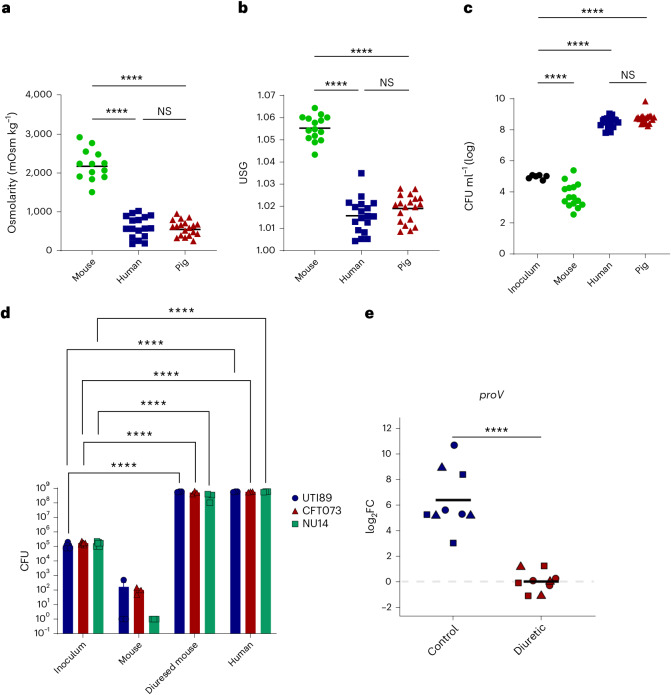
Mouse urine inhibits the growth of UPEC*.* **a**,**b**, Urine osmolarity (**a**) and USG (**b**) of mice (*n* = 18), humans (*n* = 19) and pigs (*n* = 15). **c**, CFUs per milliliter after overnight incubation of UTI89 in urine from mice, humans and pigs. **d**, The UPEC strains UTI89, CFT073 and NU14 were all significantly reduced in colony counts after 18 h incubation in pooled mouse urine (*n* = 3 for each strain) compared with inoculum (*P* < 0.0001). After incubation in human urine, and in pooled mouse urine from diuretic mice, all strains showed significant growth compared with inoculum (*P* < 0.0001). **e**, All strains significantly upregulated the osmo-responsive gene *proV* during growth in mouse urine compared with growth in urine from diuretic mice (*P* < 0.0001). Bars and horizontal lines indicate means. Statistical analysis was performed using one-way ANOVA (**a**–**c**), two-way ANOVA (**d**) and Mann–Whitney test (**e**). ****P < 0.0001. NS, not significant.

A plausible explanation for UPEC’s attenuated infectious potential in mice is that hyperosmolar urine inhibits growth. To test this hypothesis, urine specimens were collected from control mice, inoculated with UTI89 and cultured overnight. Growth experiments in urine specimens from human volunteers and pigs were conducted in parallel. Mouse urine osmolarity was outside the range of both humans and pigs (Fig. 2a,b). While UTI89 proliferated in human and pig urine by 1,000- to 10,000-fold (no significant difference between these two species), the bacterial population was significantly reduced in mouse urine by an average of 10-fold (*P* = 0.002, one-way analysis of variance (ANOVA) with Tukey’s multiple comparisons test) (Fig. 2c). Two other UPEC strains (NU14 and CFT073) were also growth-inhibited in mouse urine, thus demonstrating that UPEC strains are substantially challenged in the urinary tract of mice, contrary to humans and pigs (Fig. 2d). Furthermore, when incubating the three strains in urine collected from diuretic mice, we found growth rates equal to growth in human urine (Fig. 2d); proV expression was also downregulated in diuretic mouse urine compared with control mouse urine (Fig. 2e).

Our study demonstrates that the hyperosmolar urine of the mouse promotes UPEC filamentation, a widely accepted hallmark of UTI in humans^3,7,8^. This result aligns with earlier studies using human urine, which showed that filamentation is dependent on—or induced by—urine at the higher end of the normal range of osmolarity^9–11,17^. Reducing mouse urine osmolarity to levels corresponding to intermediate human urine osmolarity almost completely abolished bacterial filamentation, leading to increased bacterial infectious potential and survival in the mouse urinary tract.

This association aligns with findings from experimental studies in pigs, an animal with human-equivalent urine osmolality, where UPEC does not exhibit a filamentous growth response^18^. Furthermore, in contrast to results in the mouse model, recent genome-wide analyses of UPEC have revealed that osmotic stress-related genes were not fitness factors in pigs^19^. This pattern is also reflected in transcriptomic comparisons between human and mouse UTIs, which, despite reporting an overall correlation in bacterial gene expression profiles, identified hundreds of genes that were differentially expressed, including increased expression of *proV* during mouse infection^20^.

A potential limitation of the mouse diuresis model is that urine osmolarity may not be the only factor impacted by the intake of water with 20% glucose. Although we found that pH, glucose, albumin and total protein excretion were unaffected by this procedure (Supplementary Fig. 1), we cannot rule out that other urine properties such as metabolites or metal ions could be affected. In this regard, it should be mentioned that, although filamentation correlates with high overall urine solute concentration, studies with human urine indicate that specific low-molecular constituents are responsible for this phenotype rather than general osmolarity, and that pH also influences filamentation^9,11^. These specific factors were not further investigated in the current study. Furthermore, if filamentation occurs in the diuretic mice far outside the typical filamentation window in mice (12–20 h), we would not have captured it by relying on one time point (18 h). Nevertheless, our findings highlight the problem that the unique properties of mouse urine strongly influence UTI pathogenesis and UPEC survival.

The influence of hyperosmolar mouse urine on infectious potential and UTI pathogenesis highlights the need for vigilance among researchers working in the UTI field. To our knowledge, this factor has not been accounted for in previous mouse UTI studies and may have affected the interpretation of results, including the modeling of the widely accepted UTI pathogenic cascade^8^. In addition, the inhibitory effect of mouse urine on UPEC should be accounted for in preclinical evaluation of drugs and vaccines against UTI.

UTI vaccines, which are currently pursued by several companies worldwide, may show an overestimated efficacy in mice, as shown here, as bacteria are already challenged by a toxic urine environment. Regarding antibiotic susceptibility, β-lactam antibiotics—typically used against UTIs—target bacteria in a state of active cell division and may therefore have reduced activity against filamentous UPEC in mice. Indeed, studies have shown that UPEC survive β-lactam treatment in mice, but not in pigs^18^; however, the exact influence of urine osmolarity on the antibiotic activity remains to be explored.

In conclusion, the discrepancies between human and mouse urine need to be carefully considered when designing basic research studies and preclinical challenges in the murine UTI model. The model can be useful for certain studies, and its limitations can be partly circumvented by using diuretic mice, as demonstrated here. However, studies on complex UTI pathogenesis and drug activity may require the use of animals more similar to humans.

## Methods

### Ethics statement

This project was approved by the Danish Animal Experiments Inspectorate, license number: 2022-15-0201-01257. The experiments were performed according to the European Union Directive 2010/63/EU on the protection of animals used for scientific purposes.

### Strains and media

We used the *E. coli* cystitis isolate UTI89 carrying the plasmid pMAN01 which was derived from a pSC101 plasmid with a constitutively active promoter driving green fluorescent protein expression^10^. Strains were maintained using 30 µg ml^−1^ chloramphenicol in agar plates and lysogeny broth (LB) when preparing the inoculum. The bacteria were cultured to optimize type-1 fimbrial expression. In short, a colony from an agar plate was suspended in 25 ml of LB and incubated for 24 h at 37 °C. From here, 100 µl was suspended in 25 ml of fresh LB medium and incubated similarly. On the day of the infection, the broth was centrifuged at 5,000*g* for 20 min, and the pellet was resuspended in saline and adjusted to an optical density of 1.0. The bacterial concentration was validated by plating of serial dilutions and counting CFU reaching 10^9^ CFU ml^−1^. For the in vitro experiments, we used the UPEC reference strains UTI89, NU14 and CFT073, which were cultured and prepared as explained above.

### Animals used for infection

We used 117 8-week-old female C3H/HeN mice (Janvier), which were housed in the animal facility of the University of Southern Denmark and allowed 7 days of acclimatization. The animals were used as follows: analysis of USG (*n* = 68), bladder CFU (*n* = 32), kidney CFU (*n* = 56), urine CFU (*n* = 38), confocal laser scanning microscopy (CLSM) (*n* = 56) and qPCR (*n* = 24). Not all analyses could be performed from each mouse, that is, bladders could not be used for CLSM, CFU quantification, urine USG analysis and qPCR at the same time owing to insufficient biological material. However, all animals could be used for USG analysis before infection. At least 12 animals were used in each analysis per group. The mice were housed in communal cages (four animals per cage) with sawdust bedding and various enrichment. They were fed a standard diet and allowed free access to water.Experiments were performed with 20–25 mice per round and repeated until statistical significance was achieved, with the primary outcome being CLSM microscopy (see below). Cages were randomly allocated to either group. Confounders were not controlled for; K.S. was aware of group allocation during the entire study.

### Murine infection

Thirty hours before inoculation, mice were given water containing 20% glucose (w/w) to increase diuresis. Control mice were given water without glucose. The water intake per cage was registered daily. All animals were inoculated with 50 µl of a bacterial suspension by transurethral catheter as described by Hung et al.^3^. Mice were anesthetized with a mix of intraperitoneal ketamine (60 mg kg^−1^) and xylazine (12 mg kg^−1^).

Before inducing anesthesia, a urine collection attempt was performed to empty bladders and ensure equal basis of infection. Eighteen hours after infection, a new urine collection attempt was performed, and the animals were euthanized by cervical dislocation. Whole bladders were removed and bisected. One half was prepared for CLSM, and the other half was placed in 1 ml saline and kept on ice for subsequent bacterial quantification. For 24 animals, the other half bladder was used for qPCR. Both kidneys were removed and placed in 1 ml saline and kept on ice. Organs were homogenized for 10 s on ice using a rod disperser and plated in serial dilution on LB agar (SSI Diagnostica). Plates were incubated overnight at 35 °C.

For the susceptibility experiments, the UTI89 wild-type strain was used, and the animals were sedated with isoflurane. Successful infection was determined as detectable levels of UTI89 in bladder homogenates 18 h after inoculation (detection limit of 10 CFU per bladder).

### Microscopy

Bladder halves from each animal were splayed on silicone pads and fixed in 3.7% paraformaldehyde for 45 min. Hereafter, the bladders were washed three times in sterile saline and kept at 5 °C overnight. The next day, the splayed, fixed bladders were placed on glass slides and one drop of Dako Mounting Medium (Agilent Tehnologies) was added. To ensure horizontal placement of the coverglass on the heterogeneous surface of splayed bladders, a thin silicone frame was added to provide support for the coverglass. Hereafter, the specimens were subjected to CLSM using an Olympus FV1000MPE microscope. Using a ×20 objective (XLUMPlanFI ×20/0.95 W objective), the bladders were screened by an experienced researcher (T.E.A.) who was blinded to the group allocations, and the presence of bacterial filamentation was scored on a scale from 0 to 4, with 0 being no filamentation (that is, only rod-shaped bacteria) and 4 being substantial filamentation with no rod-shaped bacteria. In 16 cases, too few bacteria were observed to allow reliable scoring, and hence these animals were excluded.

### Urine collection and analysis (in vivo infection)

Urine was collected by holding the mouse over a sterile Petri dish (diameter 10 cm) and gently tabbing the abdomen. This led most animals to spontaneously release one to two droplets of urine. USG was measured at room temperature using a digital refractometer (UG-α, Atago). Urine aliquots of 10 µl were serial diluted and plated as explained above for bacterial quantification. Urine aliquots of 10 µl were added to the glucose spot on a urine dipstick test (Roche Compur 7) to assess potential urinary secretion of glucose. In one mouse, sugar was detected, and the animal was excluded from the study. Osmolarity was determined using an osmometer (Osmomat 3000, Gonotec).

### In vitro growth assay

Mouse urine was collected from four mouse breeds (BALB/c, NDMI, B6 and CD1) and pooled into 15 batches with at least 14 mice per batch (not mixing breeds or sex). Mouse urine was pooled to ensure adequate volume for sterile filtration, and measurement of osmolarity. The urine was collected from healthy mice from the animal facility of the University of Southern Denmark. Human urine specimens (*n* = 18) were collected from nine healthy volunteers (four men and five women). Pig urine (*n* = 19) was collected from eight healthy female pigs (Danish Landrace × Yorkshire) that were housed as part of another study with a different primary purpose. All urine samples were sterile filtered (pore size 0.2 µm) and stored up to 24 h at 5 °C before the experiment. Urine aliquots of 900 µl were inoculated with UTI89 (10^5^ CFU) and incubated for 21 h at 37 °C without aeration. Colony counts were quantified by plating urine aliquots on LB agar.

For comparison between UPEC strains, urine was collected and pooled from 24 mice (female C3H/HeN) and inoculated with UTI89, NU14 or CFT073 as described above. A week later, the same mice were given 20% glucose water, and the experiment was repeated for comparison.

### RNA extraction

Extraction of RNA from infected mice bladders was carried out by bead beating, followed by a phenol–chloroform extraction. Snap-frozen bladder halves were transferred to BeadBug tubes with 0.1-mm zirconium beads (prefilled tubes, 2.0 ml, Sigma-Aldrich) and RNA lysis buffer (4 M guanidinium thiocyanate, 0.1 mM Tris–HCl (pH 7.5), 10 mM sodium acetate (pH 4.5), 25 mM EDTA, 0.1 % Triton X-100 and 2 mM dithiothreitol). Bead beating with a Bead Ruptor Elite (OMNI International) was carried out at 6.5 m s^−1^ for 2× 30 s. The tubes were subsequently spun down at 10,000*g* for 2 min at 4 °C, and the supernatant was transferred to tubes with 700 μl acidic phenol (pH 4.5) and 300 μl chloroform. Tubes were then inverted and incubated at 80 °C for 4 min, followed by cooling on ice. Subsequently, tubes were centrifuged at 14,100*g* for 5 min, and the aqueous phase was transferred to 96% ethanol with sodium acetate (37.5 mM) and precipitated overnight. RNA was pelleted by centrifugation (20,000*g* for 45 min at 4 °C) and washed in ice-cold ethanol. RNA pellets were resuspended in RNase-free water and stored at −80 °C. RNA from UPEC strains cultured in vitro in mouse urine was extracted directly from lysed pellets using phenol–chloroform extraction, without bead beating.

### RT–qPCR

RT–qPCR was carried out on RNA extracted from UPEC-infected mice bladders, or from UPEC strains cultured in vitro in mouse urine. For the in vitro cultures, LB was inoculated with UTI89, NU14 or CTF073 and incubated at 37 °C until an optical density at 600 nm (OD_600_) of 1.0. The bacteria were then pelleted, resuspended in equal volumes of urine pooled from 24 female C3H/HeN mice, and incubated for 1 h at 37 °C. A total of 1 µg RNA was treated with 0.2 units RNase-free DNase I (New England BioLabs) in 1× DNase I Reaction Buffer before cDNA synthesis was performed using the High-Capacity cDNA Reverse Transcription Kit (Applied Biosystems, Thermo Fisher). Real-time qPCR of cDNA samples was carried out with RealQ Plus 2× Master Mix Green without ROX (Ampliqon) using supplier-recommended PCR settings on a CFX Opus 384 Real-Time PCR System (Bio-Rad). Relative changes in gene expression were calculated using the ΔΔCT method using gyrA for normalization. The following primers were used for the RT–qPCR analysis: ompC_F (5′-GGCCAGTGGGAATATCAGATCC-3′), ompC_R (5′-CACCGAATTCTGGCAGTACG-3′), proV_F (5′-TTGCGATGGTCTTCCAGTCC-3′), proV_R (5′-CCCGCCAGAGAGTTCATCC-3′), gyrA_F (5′-GCGGTTTATGACACGATCGTC-3′) and gyrA_R (5′-AGATCAGCCATCAGTTCATGGG-3′).

### SDS–PAGE

Urine samples were mixed with NuPAGE LDS sample buffer (×4) and NuPAGE Sample Reducing Agent (×10) and separated on 4–12% Bis-Tris NuPAGE gels in MOPS running buffer using the NuPAGE system (Invitrogen). Coomassie staining was performed by incubating the gels with Novex SimlyBlueTM SafeStain (Invitrogen) and destained overnight in H_2_O. The gel was imaged by Molecular Imager ChemiDocTMXRS+ (Bio-Rad).

### Urine analysis

Albumin and creatinine in spot urine samples were detected by a commercial sandwich ELISA kit (E99-134, Bethyl Laboratories), and a colorimetric detection kit (KGE005, R&D systems) according to manufacturer’s protocol, respectively. Urine pH and osmolarity were measured with Lab pH Meter (PHM220, MeterLab) and Osmomat300 (Gonatech), respectively.

### Statistics

Statistical analyses were performed using GraphPad Prism Software (version 9.3.1). The nonparametric Mann–Whitney test was used for comparison of two groups. One-way ANOVA was used for comparison of more than two groups with normal distribution with Tukey’s multiple comparisons test. The Kruskal–Wallis test was used for comparison of more than two groups of nonparametric data with Dunn’s multiple comparisons test. Contingency tables were analyzed for significance using Fisher’s exact test. ****P < 0.0001, ***P < 0.001, **P < 0.01, *P < 0.05. *P* values below 0.05 were considered significant. Statistical tests were two-sided, and all measurements were taken from distinct samples.

### Reporting summary

Further information on research design is available in the Nature Portfolio Reporting Summary linked to this article.

## Online content

Any methods, additional references, Nature Portfolio reporting summaries, source data, extended data, supplementary information, acknowledgements, peer review information; details of author contributions and competing interests; and statements of data and code availability are available at 10.1038/s41684-026-01727-4.

## Supplementary information

Supplementary Information Supplementary Fig. 1 and legend.

Reporting Summary

## Data Availability

The data that support the findings of this study are available from the authors upon request.
