# Supplementary material for: Hyperosmolarity of mouse urine confounds research in urinary tract infection
Source: Lab Anim (NY). 2026 Apr 29;55(7):267–70. doi: 10.1038/s41684-026-01727-4 (PMC13318629; doi:10.1038/s41684-026-01727-4)
Supplement: Supplementary file 1 — Supplementary Fig. 1 and legend. [file 41684_2026_1727_MOESM1_ESM.pdf]

https://doi.org/10.1038/s41684-026-01727-4

Hyperosmolarity of mouse urine confounds research in urinary tract infection

In the format provided by the authors and unedited

Supplementary information

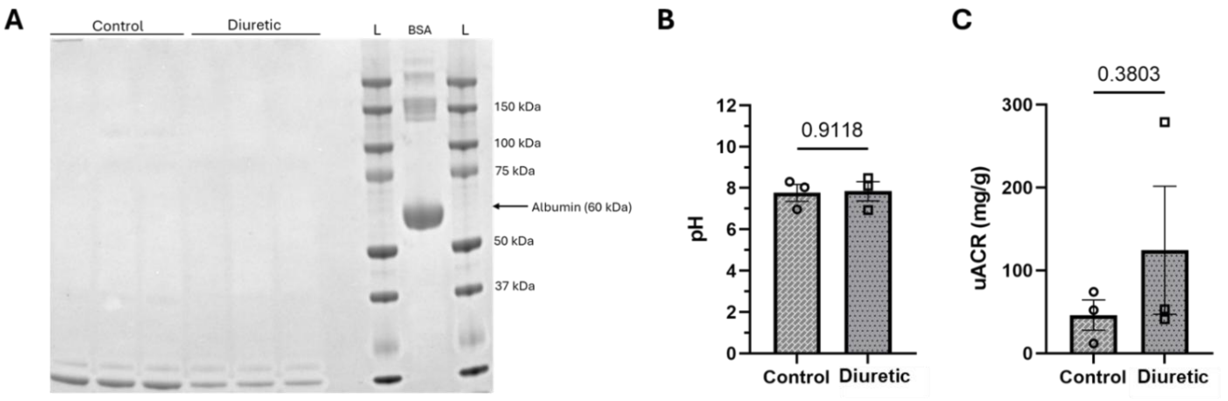

Supplementary figure 1. Urine properties. A. SDS-PAGE gel separation and coomassie protein staining of creatinin-adjusted (8 mg/dL) showed no visible proteinuria in either control mice or mice supplemented with 20% glucose water, demonstrating no induced proteinura as a consequence of renal overload. Bovine serum albumin (BSA) (10 mg/mL) was used as positive control for albumin. B-C. Similarly, urine pH and urine albumin-creatinine ratio (uACR) were unaffected by the procedure. L, Ladder
